# Mandibular Reconstruction after Resection of Ameloblastoma by Custom-Made CAD/CAM Mandibular Titanium Prosthesis: Two Case Reports, Finite Element Analysis and Discussion of the Technique

**DOI:** 10.3390/dj11040106

**Published:** 2023-04-20

**Authors:** Antonio Cortese, Francesca Spirito, Pier Paolo Claudio, Lorenzo Lo Muzio, Alessandro Ruggiero, Maurizio Gargiulo

**Affiliations:** 1 Unit of Maxillofacial Surgery, Department of Medicine, Surgery, and Dentistry, University of Salerno, 84084 Salerno, Italy; 2 Department of Clinical and Experimental Medicine, University of Foggia, 71122 Foggia, Italy; 3 Department of Pharmacology and Toxicology, Cancer Center & Research Institute, University of Mississippi Medical Center, Jackson, MS 39216, USA; 4 Department of Maxillofacial Surgery, Cancer Center & Research Institute, University of Mississippi Medical Center, Jackson, MS 39216, USA; 5 Department of Industrial Engineering, University of Salerno, Via Giovanni Paolo II, Nr. 132, 84084 Fisciano, Italy; 6 U.O.C. Chirurgia Maxillofacciale, A.O.R.N. “A. Cardarelli”, Via A. Cardarelli, 80131 Naples, Italy

**Keywords:** ameloblastoma, CAD-CAM, reconstruction, titanium, prosthesis, mandible

## Abstract

Virtual surgical planning for CAD/CAM mandibular reconstruction by titanium prosthesis was recently reported for resected cases. Even if some advantages are evident, difficulties that may arise for TMJ function after reconstruction originate from prosthesis contamination through oral mucosa dehiscence. In these two cases reported of mandibular reconstruction after resection of ameloblastoma by custom-made CAD/CAM titanium prosthesis, the procedures were aimed to preserve the TMJ glenoid cavity and articular disc avoiding functional problems for hemi-mandibular resections that included the condyle (as in case #1) or with condylar preservation (as in case #2) and avoiding intraoral incisions in both cases. The entire surgical planning and prosthetic fabrication were explained with specifications and the sequence of the surgical procedure. Finite elements analysis (FEA) was performed to check the force distribution and efficacy of the prosthetic device (case 1 with hemi-mandibular resection and rehabilitation). Although successful in these two cases, surgical reconstruction of the mandibular defect after resection by a CAD-CAM custom-made prosthesis still shows some drawbacks and failure risks. Several advantages of this technique and the surgical success in these two cases were presented, but limitations and side effects must be considered when cases are selected.

## 1. Introduction

According to the World Health Organization definition, ameloblastoma is a benign intraosseous progressively growing epithelial odontogenic neoplasm characterized by expansion and a tendency for local recurrence if not adequately removed. It represents the second most common odontogenic tumor after odontomas, and it is mostly diagnosed in the fourth and fifth decades. According to the clinical, histological, and radiographic characteristics, it could manifest as a uni-cystic, multi-cystic, peripheral, adenoid, and metastasizing form [1,2,3].

The treatment strategy for ameloblastoma consists of either resective or non-resective surgical therapy. It is possible, for surgical treatment, a conservative or a radical approach [4]. Conservative surgical treatments include enucleation with cauterization, curettage, cryotherapy, or marsupialization. The conservative approach has some advantages such as the preservation of the patient’s healthy tissues, the reduction of aesthetic dysfunctions, and a better quality of life after surgery. A radical surgical approach is a choice in the case of biologically aggressive subtypes of primary and recurrent ameloblastoma and it is based on en-bloc tumor resection with wide bone margin followed by immediate or delayed reconstruction of the surgical defect [5].

Non-surgical management of ameloblastoma consists of radiotherapy, chemotherapy, or target therapy and it is addressed to patients medically unstable for surgery or to treat ameloblastic carcinoma and recurrent ameloblastoma after multiple postsurgical recurrences [4,6,7].

Risks for malignant transformation after radiotherapy have been reported [4].

Ameloblastoma is characterized by a high recurrence rate related to surgical treatment strategy, indeed incidence of recurrence ranges from 55% to 90% following conservative treatment, significantly higher than the incidence rate of 15–25% following more radical treatment. However radical treatment strategy is associated with a significantly higher risk of post-surgical complications, a lower rate of prosthetic rehabilitation, and serious aesthetic and functional impairment [8,9].

After radical surgery, the restoration of function and acceptable aesthetics represent the primary goals of reconstructive surgical procedures. Several options for reconstruction of the bone defect have been described: vascularized or not vascularized bone grafts, bone chip grafts from the iliac crest and conformed custom-made grids in association with bone morphogenic protein, and finally, distraction osteogenesis [10,11,12]. Involvement of the temporomandibular joint (TMJ) adds another level of complexity since a functional and stable joint must also be reconstructed to achieve efficient jaw movement and mastication [13].

The pedicled flap with or without a titanium reconstruction plate is the technique most often used for mandibular reconstruction [12], but several limitations have been reported such as the establishment of a new surgical field, plate exposure or fractures, TMJ complications, and possible esthetic dysfunction [14,15]. Recently, virtual surgical planning for CAD/CAM mandibular reconstruction with three-dimensional (3D) custom-made titanium prosthesis has been reported for resected cases and represents an alternative in those cases with free flap contraindications or refusal by the patient [16].

This reconstruction technique using CAD/CAM technology has several advantages such as visualization of tumor margins, the definition of surgical margins, fabrication of surgical templates and cutting guides, evaluation of continuity defects, identification of ideal plate dimensions and shape for reconstruction, reduction of surgery time and the fabrication of custom-made prostheses [17]. The main difficulties may arise from the correct management of TMJ function after reconstruction or from prosthesis contamination through oral mucosa dehiscence.

To investigate the accuracy and effectiveness of various reconstruction approaches, a finite element analysis (FEA) can be performed [18].

FEA is a valuable tool for stress investigations due to its simplicity. It is a numerical method for breaking down complex geometries into a large number of simple domains (elements) linked by nodes. The tensions and deformations in these simple elements can be evaluated at each node once the geometrical model has been subdivided to evaluate the stress and strain of important structures [19].

Reconstructing mandibular defects with titanium prosthesis using FEA support appears to reduce the risk of screw pull-out and plate fractures [20].

The use of FEA also has several utilities in oral rehabilitation, in fact, in many recent studies FEA was adopted as a method to check the advantages of different implant treatment options for edentulous mandibles from a biomechanical standpoint. In recent reports, maximum stress was detected for cantilever and 45° tilted implants by FEA test of the all-on-four prosthesis [21]. Analysis of the stress forces at the implant–bone interface was detected, preventing significant effects on biological bone resorption leading to implant failures [21,22,23].

Recent studies stated that stiff materials reduced stress on implants; also, short implant adoption could avoid cantilevers and tilted implants in atrophic mandibles [24,25,26].

By describing these two case reports, we aim to illustrate the procedure we followed for reconstructing a large bone defect after mandibular resection for ameloblastoma treatment, using a 3D custom-made titanium prosthesis, also performing a FEA to check force distribution and efficacy of the prosthetic fabrication, preserving the TMJ glenoid fossa and articular disc to avoid functional problems.

## 2. Case Reports

Informed consent was obtained from all the patients included in this study and the research was approved by the Ethical committee of the University of Salerno (Ethical approval number: n.40 (4 April 2019).

### 2.1. Patient #1

**Figure 1:**
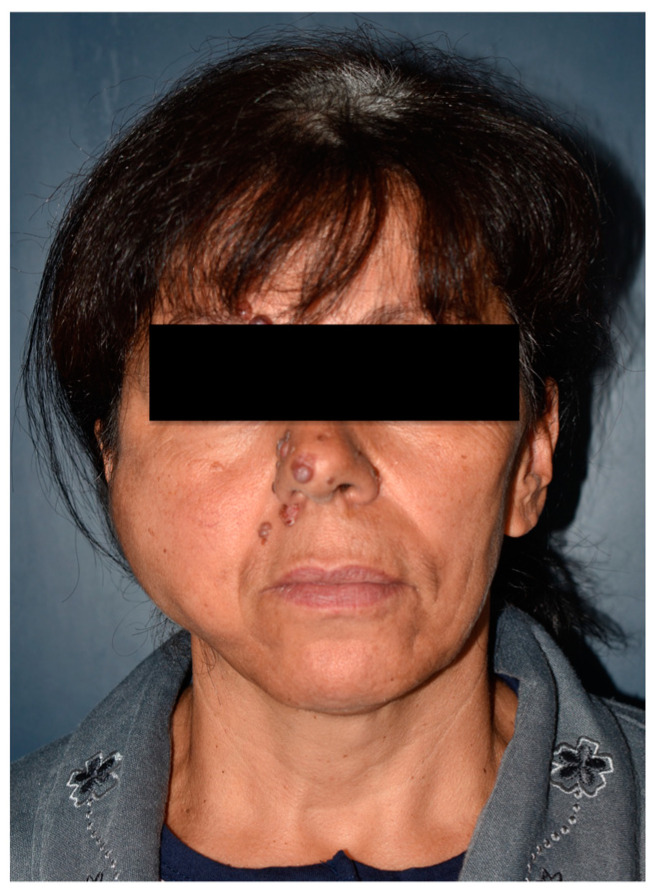
Pre-surgical frontal clinical view of the extraoral asymmetry of the lower third of the face.

A 54-year-old female patient came to our attention in October 2019 at the Maxillo-Facial Unit of the University Hospital of Salerno for the management of swelling in the right side of the mandible (Figure 1).

The patient referred that she received previous multiple operations for ameloblastoma of the left mandibular side performed in other hospitals, with the first operation in 1977, and the second and third operations for relapses in 1980 and 1999. Clinical documentation was not available, and the patient could not describe the kind of surgical operations received.

**Figure 2:**
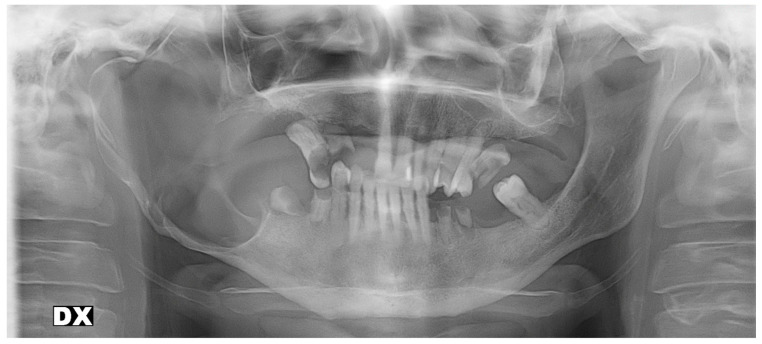
Pre-surgery panoramic X-ray showing a wide radiolucent lesion extending from dental elements 4.5 to the entire right mandible ramus involving the right mandibular body.

**Figure 3:**
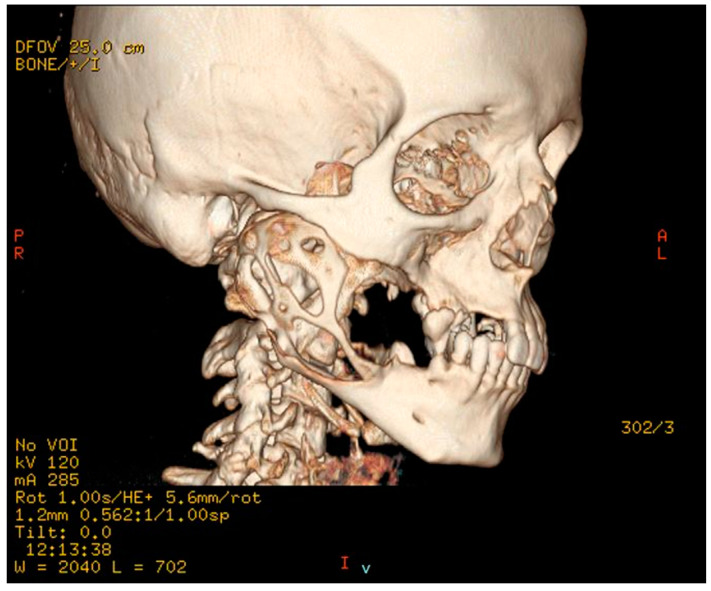
CT scan 3D reconstruction showing the huge lesion extending from dental elements and 5 to the entire right mandible ramus involving the right mandibular body and the alveolar nerve canal, with perforations of the cortical bone wall on both buccal and lingual sides.

Clinical examination revealed evident extraoral asymmetry of the lower facial third and an intraoral buccal-lingual expansile lesion in the right mandible. Radiographic examination by Rx panoramic and CT scan showed a wide radiolucent lesion extending from dental elements 4.5 to the entire right mandible ramus involving the right mandibular body and the alveolar nerve canal, with perforations of the cortical bone wall on both buccal and lingual sides (Figure 2 and Figure 3).

For the radiologic characteristics seen on the CT imaging, a diagnosis of invasive and destructive ameloblastoma was made.

The treatment plan included a right-side mandibulectomy with 8 mm safety margins and the reconstruction of the affected portion of the mandible with a fibular free flap for microsurgical reconstruction or microsurgical bone graft from other sites such as the iliac crest or scapula. However, the patient refused every reconstructive surgery that involved a second surgical site and therefore impairment due to microsurgical flap elevation at the donor site. Therefore, these reconstructive hypotheses were discarded, and alternatives were explored. Among these, a bone resection alongside a reconstruction by modern 3D techniques applying a CAD-CAM digitalized and customized titanium prosthesis for precision surgery was the solution that best suited the functional and aesthetic patient’s requests.

### 2.2. Design Case #1

Adequate maintenance of the midline, occlusion, and mandibular dynamics was all achieved through digital processing of TC cone beam, following the acquisition of 3D images to define the resection lines with safety margins and a CAD-CAM procedure to obtain the cutting guides and a prosthesis with the correct dimensions. 

After the intervention was simulated and planned on 3D models obtained from the CT scans (Figure 3), mandibular osteotomy surgical guides that allowed a precise resection were constructed. Resection lines were achieved through precise surgical guide positioning by fitting with the mandibular symphyseal bone contour. The fixation screws pre-holes were constructed from the surgical guide data to facilitate the accurate fitting of the CAD-CAM custom-made prosthesis.

**Figure 4:**
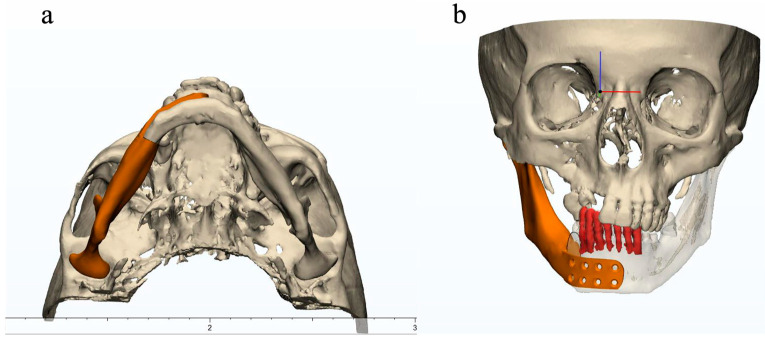
Simulation of the general shape of the mandibular prosthesis delineated from the mirrored image of the unaffected side on 3D models. (**a**) inferior view; (**b**) frontal view.

Because of the expansile presentation of the lesion with subsequent deformity, mirroring of the unaffected side of the mandible was performed, and the general shape of the mandibular prosthesis was delineated from this mirrored image (Figure 4a,b).

Using additive manufacturing technology 3D plastic prototypes (pre-surgery model, sectioned mandible model, and mandibular prosthesis prototype) were manufactured.

After the approval of the prototypes, the mandibular prosthesis was made with SLM (selective laser melting) technology, i.e., with a production process by adding laser-stratified titanium powder instead of extrusion from a single block of titanium, thus obtaining a structure with controlled degree of rigidity. This production feature reduces the so-called stress shielding, which is the altered distribution of loads between the prosthesis and the bone. The prosthesis also presented a trabeculation of the internal structure to lighten the weight and ensure better osteoinduction.

**Figure 5:**
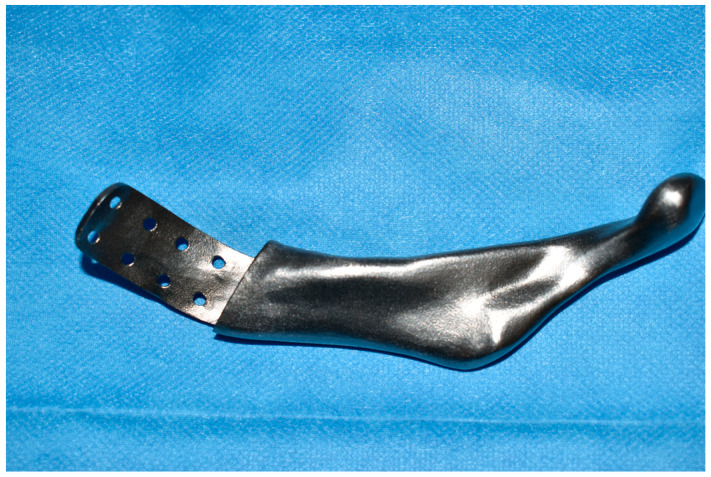
CAD-CAM custom-made hemi-mandibular prosthesis.

The prosthesis shape was manufactured by an accurate procedure able to precisely fit with the symphyseal resection stump and was anchored with 8 bicortical screws in the predrilled holes of the prosthesis, with a wing overlapping the symphysis. Bicortical screw fixation was adopted to avoid tilting inside the wing plate holes (Figure 5). 

### 2.3. Finite Element Analysis Case #1

**Figure 6:**
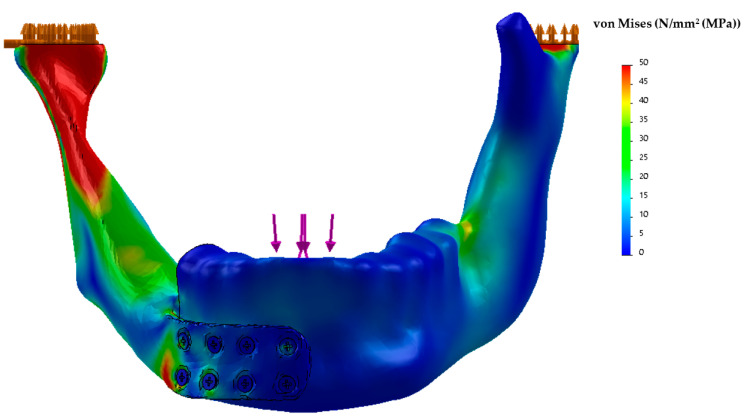
Finite element analysis (FEA) showing von Mises stress concentration on the prosthesis on the conjunction margin of the plate with the first lower right screw (the peak stress was 50 MPa) and on the interior surface of the condylar portion of the plate (the peak stress was 50 MPa). FEA was conducted assuming a static load of 600 N on the central incisors (purple arrows) also resulting in an upward load on both condylar surfaces for elevator muscles action (orange arrows).

Using CT data, a finite element analysis (FEA) of the implant was performed by bioengineers to identify potential causes of mechanical failure (Figure 6). The main areas of von Mises stress concentration on the prosthesis were: (A) the conjunction margin of the plate with the first lower right screw (the peak stress was 50 MPa); (B) the interior surface of the condylar portion of the plate (the peak stress was 50 MPa). Both areas were adequately supported by prosthesis thickness.

Because of the undercuts and the trabeculated hollow structure of the mandibular prosthesis, selective laser melting was adopted for manufacturing concerning technical advantages in comparison to the milling technique. 

Other biomaterials have not been simulated or considered for manufacturing this kind of device because medical titanium Ti6Al4V ELI grade 5 ASTM F136 realized according to SLM (selective laser melting) technique is the most common biomaterial for skeletal prosthesis according to the current literature.

Among metallic biomaterials such as stainless AISI 316L steels and Co–Cr alloys, titanium, and its alloys exhibit the most suitable characteristics for biomedical applications, because of their high biocompatibility, specific strength, and corrosion resistance [27,28]. 

Titanium and its alloys are currently adopted in implants such as artificial hip joints and dental roots and in all cases in which implants replace hard tissue. They are required to have high strength and long fatigue life, that is, high fatigue strength. Nowadays, a low Young’s modulus equivalent to that of cortical bone is simultaneously required in order to inhibit bone absorption.

The stress/strain FEM analysis was conducted assuming a load similar found in the article by Dahake et al. [29]. In particular, the static structural load conditions were applied for analysis; the load condition was added by considering a force of 600 N perpendicular to the occlusion surface added on the central incisors. The properties of the material used in the simulations are the following for the different parts of the model: mandible (cortical bone): Young’s modulus 8700 MPa, Poisson’s ratio: 0.28, tensile strength 85 Mpa; titanium screws and titanium structure: Young’s modulus 105,000 MPa, Poisson’s ratio: 0.3, tensile strength 897 Mpa; frictional coefficient: 0.3 between the framework and screws, implant fixation plate and bone, implant side and bone, as it was previously described [30]. The software used for FEA was Abaqus by SIMULIA.

### 2.4. Surgical Sequence Case #1

**Figure 7:**
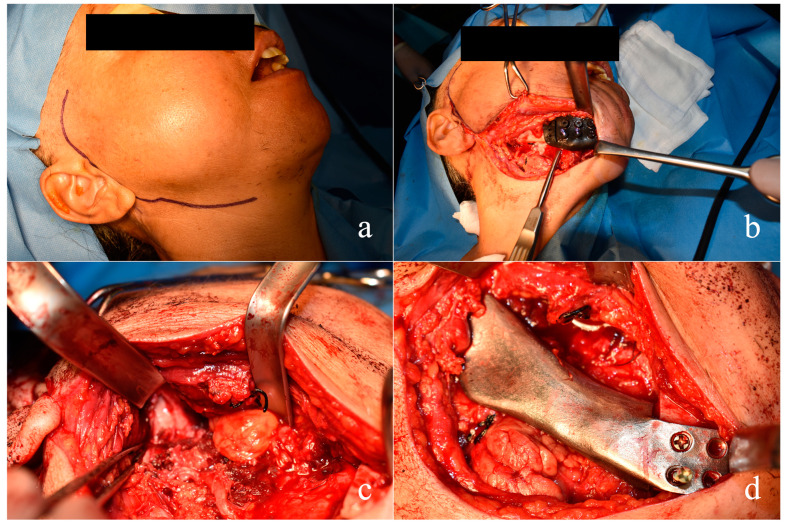
Intra-operative sequence: (**a**) intra-operative view: trans-cutaneous access in the submandibular area. (**b**) Positioning of the custom-made surgical guide. (**c**) Preservation of the articular disc. (**d**) Positioning of the custom-made mandibular prosthesis.

The resection and reconstruction procedures were carried out to preserve the integrity of the mucosa, avoiding any perforation and any transmucosal device. The operation was carried out completely by trans-cutaneous access in the submandibular area for better aesthetic results of the surgical scar (Figure 7a).

The incision was carried out following a sliding surgical plane technique performing an incision of the cutaneous, subcutaneous, and muscular plane in different sites connected by stepwise dissections to obtain a wider regenerating supply with larger tension distribution and better healing results (as already described in our previous work) [31]. 

The mandibular prosthesis was planned considering the presence in the mandible of the natural lower dental arch teeth up to 4.4 which would allow an acceptable chewing function.

Resection lines were performed following the surgical guide fitted to the mandibular symphyseal bone contour. The fixation screw pre-holes were constructed from the surgical guide data to facilitate the accurate fitting of the CAD-CAM custom-made prosthesis (Figure 7b).

Since the main problem that can occur with this kind of reconstruction by customized mandibular prosthesis is dysfunction of the TMJ and the need to implant a prosthesis also for the glenoid fossa, a radical right mandibulectomy comprehensive of the right condylar head involved by the tumor was performed. Because the articular disc was not involved by the tumor (Figure 7c), and consequently the upper compartment of the right TMJ, these structures and their related functions were also preserved. Thereafter, the prosthesis was inserted with the condyle onto the disc, by suturing the ligament apparatus and tissues around the prosthetic condylar neck (Figure 7d).

The functionality of the facial nerve with its fibers was preserved while the inferior alveolar nerve, which was entirely incorporated in the tumor, was sacrificed.

### 2.5. Results Case #1

The postoperative course was good with limited soft tissue edema and good functional recovery from the very first few days, allowing swallowing of soft foods in the early post-operative time after wound healing and chewing after 2 weeks from the operation.

**Figure 8:**
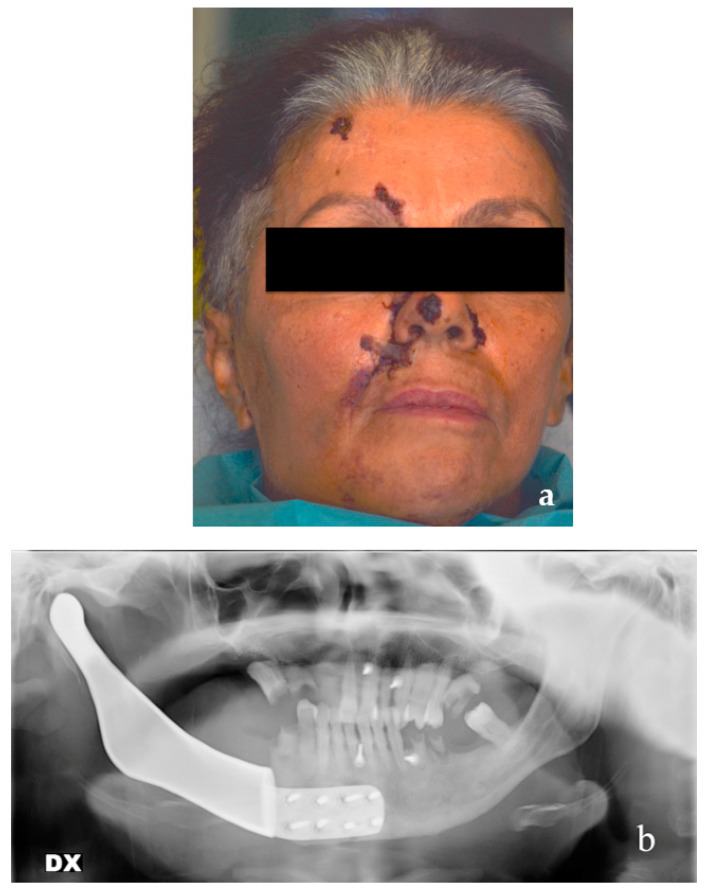
(**a**) Post-surgical frontal clinical view of the restored symmetry of the lower third of the face. (**b**) Post-surgery panoramic X-ray of the mandibular prosthesis positioning.

Figure 8 shows the postoperative clinical and radiologic results. The post-surgical frontal view of the patient with the corrected symmetry of the face and the panoramic X-ray image of the correct positioning of the mandibular prosthesis are shown in Figure 8a,b, respectively.

### 2.6. Patient #2

A 33-year-old female patient came to our attention at the Maxillo-Facial Unit of AORN “A. Cardarelli” in Naples (Italy) for the management of a jaw swelling in the right mandible side due to a recurrence of ameloblastoma.

**Figure 9:**
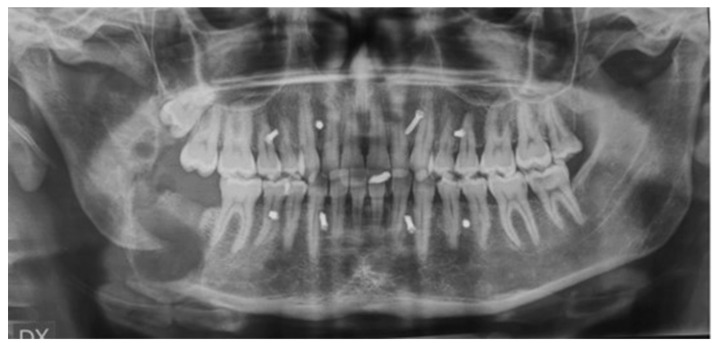
Pre-surgery panoramic X-ray showing a wide radiolucent lesion involving the body and ramus of the right mandible.

**Figure 10:**
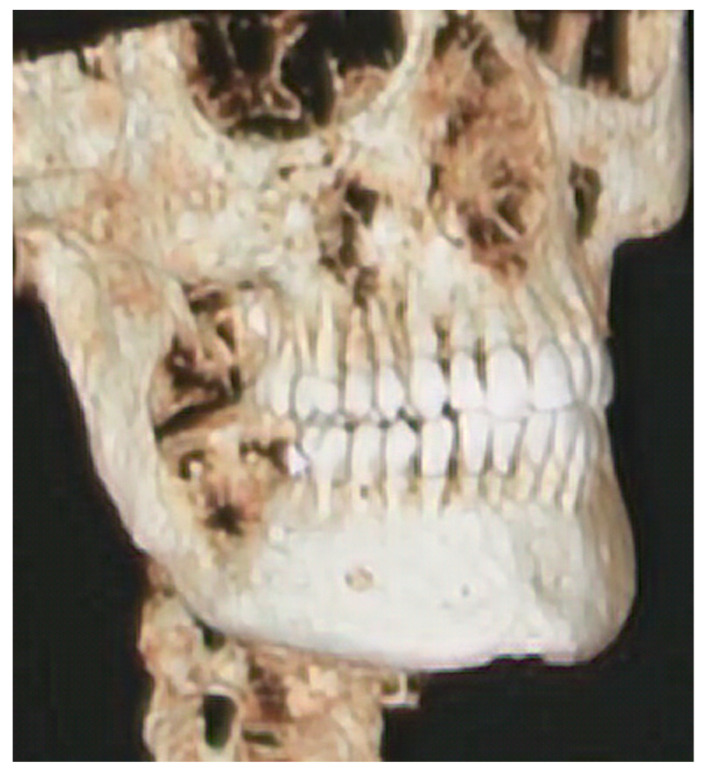
CT scan 3D reconstruction showing the ameloblastoma lesion involving the body and ramus of the right mandible.

Radiographic examination by panoramic X-ray and CT scan showed a wide osteolytic lesion with tumor invasion of the mandibular canal and the vascular-nervous bundle, with a pathological mandibular fracture due to the presence of the growing tumor (Figure 9 and Figure 10). CT scan also showed the tumor mass invading the peri mandibular soft tissues.

After the biopsy, the histopathological examination confirmed the diagnosis of ameloblastoma recurrence.

**Figure 11:**
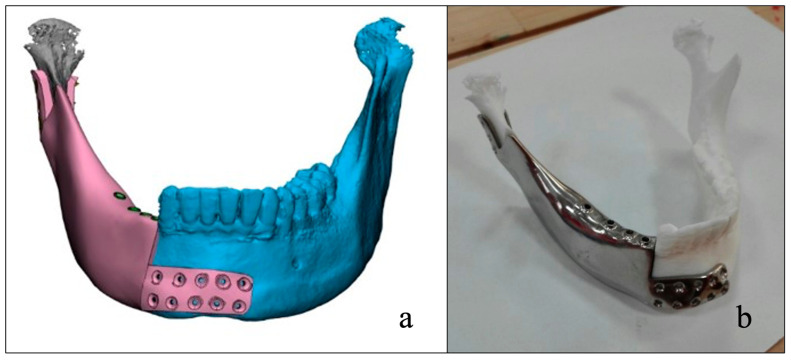
(**a**) Simulation of the general shape of the mandibular prosthesis delineated from the mirrored image of the unaffected side on 3D models in frontal view; (**b**) CAD-CAM custom-made mandibular prosthesis.

The patient refused a reconstruction with the fibula due to possible sequelae to the leg and risk for functional deficits. Therefore, the surgical planning included resection of the mandibular body and ramus preserving condyle and right symphysis region. Surgical resection was planned by virtual reconstruction and CAD-CAM custom-made osteotomy guides. Osteotomy size was planned at the sub-condylar and para-symphyseal right level; reconstruction was planned by a hollow structured titanium prosthesis smooth on the outside, and rough inside, designed and built with a CAD-CAM laser-melting technique from CT data (Figure 11a,b).

### 2.7. Design Case #2

The design and construction technique of the surgical guides and titanium prosthesis was conducted similarly to those previously described for patient #1.

### 2.8. Surgical Sequence Case #2

Surgery began with a right cervical skin incision with a curved shape at two fingers from the lower edge of the right mandibular body. A skin with a platysma flap was raised, and the dissection was carried on up to the mandibular external bone cortex with periosteal elevation for the entire bone segment with dental extractions of 44, 45, and 46. Mandibular osteotomies at the planned level (sub-condylar and para-symphyseal right area distally to 43) were performed using osteotomy CAD-CAM cutting guides designed by digital custom-made with 3D printing procedure as previously described.

Fixation was performed with 13 bicortical titanium screws (Cizeta, Italy) inserted in the prosthetic wings precisely fitting the cortical bone stumps. Autologous fat was obtained from the left abdominal subcutaneous region using a cannula, which was grafted after the Lipogems technique treatment to cover the entire mandibular prosthesis surface.

### 2.9. Results Case #2

**Figure 12:**
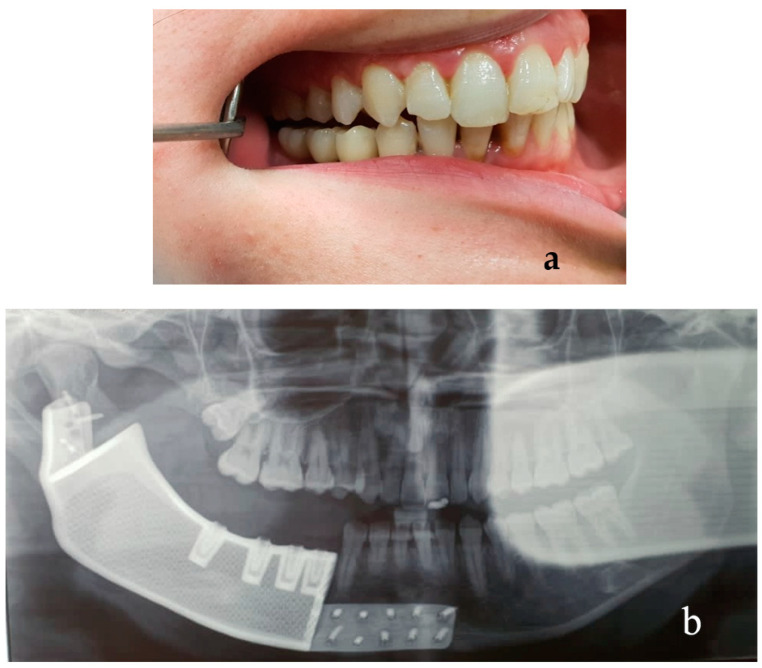
(**a**) Post-surgery view of intraoral rehabilitation. (**b**) Post-surgery panoramic X-ray of the positioned mandibular prosthesis.

In addition, the postoperative course of patient #2 was satisfactory with limited soft tissue edema and good functional recovery from the very first few days, allowing swallowing of soft foods in the early post-operative time after wound healing and chewing just after 2 weeks from the operation. Figure 12 shows the postoperative intraoral rehabilitation achieved and the radiologic results. The post-surgical intraoral view of the patient and the panoramic X-ray image of the correct positioning of the mandibular prosthesis are shown in Figure 12a,b, respectively.

Symmetry was respected and functionality was restored. The surgical wounds had optimal healing, there were no complications due to dehiscence and contamination of the mandibular prosthesis. Follow-up was of one year for each patient free from side effects or complications.

## 3. Discussion

Even today, mandible reconstruction after large resections is a challenging procedure despite the notable developments in the surgical methods available. Microvascular reconstruction with a fibula or scapula flap remains the gold standard, although these procedures have some disadvantages such as the complexity and invasiveness of the surgical technique with related risks, particularly in elderly patients, and the need for two surgical teams and prolonged post-operatively care, with a donor site causing morbidity and aesthetic relics, which are unwanted by young patients, especially if female. Prosthetic procedures can lead to suboptimal aesthetic–functional results, with problems for the survival of the implant due to limited bone graft height and the absence of keratinized gingiva.

The use of CAD-CAM technology for the fabrication of custom-made surgical guides and titanium prostheses from CT scans represents an alternative in some cases, especially in the areas of craniomaxillofacial trauma, orthognathic surgery, and reconstructive maxillofacial surgery after resection for neoplasms, which are not a candidate to postoperative radiotherapy.

In patient #1, condylar resection was needed because of tumor invasion of the condylar structure. To limit post-surgical difficulties after mandibular resection with condylotomy such as the resorption of the glenoid fossa with the displacement of the prosthetic condylar head in the skull base, we preserved the TMJ disc allowing stability in the position of the prosthetic condyle and avoiding the risk of bone resorption of the glenoid cavity. This decision was related to the tumor extension and condylar invasion detected on CT images. From the functional point of view, TMJ and condyle preservation provides paramount advantages for masticatory function and surgical procedures. In cases in which a condylar resection is needed, preservation of other TMJ structures such as the disk, muscles, and ligaments is recommended if tumor-free margins can be guaranteed.

To avoid the risks of intraoral exposure of the mandibular reconstructive prosthesis, the operation was entirely conducted transcutaneously without creating any dehiscence in the oral cavity. Furthermore, the analysis of the technical and scientific aspects was verified by bioengineers with finite element analysis (FEA) of the mandibular prosthesis, and two areas of stress concentration were identified on the conjunction margin of the plate with the first lower right screw and the anterior surface of the condylar portion of the plate. Despite the presence of these two points characterized by greater stress, the prosthesis was considered suitable for use as it would have been supported at the condylar level by the residual anatomical portion of the articular disc that we were able to maintain; furthermore, at the level of the plate margin with the screws, the stress was not significant as the bicortical positioning of the screws allowed the absence of their tilting and therefore avoided the stress amplification. Several studies have shown the paramount influence of material quality and stiffness on proper stress reduction of the bone stump and fixation screws, as evidenced by finite element analysis (FEA). In particular, it has been highlighted that stiffer framework materials concentrate more stress in their structure, reducing the magnitude of stress on the prosthetic screw [32].

Regarding patient #2, a lipofilling method was adopted using the Lipogems technique, thus avoiding the risks of intra-oral exposure to the prosthesis.

The report of these two cases of mandibular reconstruction after a large resection illustrates the use of CAD/CAM technology in the fabrication of a custom-made titanium prosthesis. Although several advantages were found by using this technique through the surgical success we observed, some limitations or disadvantages in its application must be considered that can be resolved through an accurate selection of the patients.

This reconstruction technique offers several benefits, including accurate reconstruction, decreased operative time, limited invasiveness, improved predictability of outcomes, improved patient satisfaction, and decreased complications [33]. It is a simpler and less invasive method, providing optimal and predictable aesthetic and functional results without the need for a donor site and the related risks of morbidity and unwanted aesthetic and functional consequences, especially in young and female patients. The disadvantages are mainly related to the limitations of using titanium prostheses to reconstruct surgically resected cancer patients that are candidates for postoperative radiotherapy due to the related risks of radiation exposure in the presence of metal prostheses. Additionally, the risk of dehiscence and mandibular reconstructive prosthesis exposure after prosthetic rehabilitation must be considered and carefully managed with the use of thick flaps which may include repositioning of the ipsilateral submandibular gland on the prosthesis crest.

In case of failure, a new prosthesis or microsurgical flap or a combination of a new prosthesis with bone reconstruction by microsurgical flaps or bone chips can be considered [34,35,36,37].

## 4. Conclusions

Reconstruction of the mandible after large resections remains a challenging procedure despite advancements in surgical methods. The use of CAD-CAM technology for custom-made surgical guides and titanium prostheses offers several advantages such as accuracy, reduced invasiveness, and improved predictability of outcomes. Our report on two cases of mandibular reconstruction highlights the advantages of this procedure. However, limitations and disadvantages also need to be considered, such as risks for patients who are candidates for postoperative radiotherapy and the risk of peri-implantitis after prosthesis exposure. Careful patient selection and management of potential risks are essential to guarantee optimal outcomes.

## Data Availability

Data are available upon reasonable request.
